# Neuronal Correlates of the Set-Size Effect in Monkey Lateral Intraparietal Area

**DOI:** 10.1371/journal.pbio.0060158

**Published:** 2008-07-01

**Authors:** Puiu F Balan, Jeff Oristaglio, David M Schneider, Jacqueline Gottlieb

**Affiliations:** 1 Department of Neuroscience, Columbia University, New York, New York, United States of America; 2 Department of Psychiatry, Columbia University, New York, New York, United States of America; National Institute of Mental Health, United States of America

## Abstract

It has long been known that the brain is limited in the amount of sensory information that it can process at any given time. A well-known form of capacity limitation in vision is the set-size effect, whereby the time needed to find a target increases in the presence of distractors. The set-size effect implies that inputs from multiple objects interfere with each other, but the loci and mechanisms of this interference are unknown. Here we show that the set-size effect has a neural correlate in competitive visuo-visual interactions in the lateral intraparietal area, an area related to spatial attention and eye movements. Monkeys performed a covert visual search task in which they discriminated the orientation of a visual target surrounded by distractors. Neurons encoded target location, but responses associated with both target and distractors declined as a function of distractor number (set size). Firing rates associated with the target in the receptive field correlated with reaction time both within and across set sizes. The findings suggest that competitive visuo-visual interactions in areas related to spatial attention contribute to capacity limitations in visual searches.

## Author Summary

It is well known that the brain is limited in the amount of sensory information that it can process at any given time. During an everyday task such as finding an object in a cluttered environment (known as visual search), observers take longer to find a target as the number of distractors increases. This well-known phenomenon implies that inputs from distractors interfere with the brain's ability to perceive the target at some stage or stages of neural processing. However, the loci and mechanisms of this interference are unknown. Visual information is processed in feature-selective areas that encode the physical properties of stimuli and in higher-order areas that convey information about behavioral significance and help direct attention to individual stimuli. Here we studied a higher-order parietal area related to attention and eye movements. We found that parietal neurons selectively track the location of a search target during a difficult visual search task. However, neuronal firing rates decreased as distractors were added to the display, and the decrease in the target-related response correlated with the set-size-related increase in reaction time. This suggests that distractors trigger competitive visuo-visual interactions that limit the brain's ability to find and focus on a task-relevant target.

## Abstract 3

Single-neuron activity in the lateral intraparietal area declines with the number of distractors and provides the first known neural correlate of the set-size effect in visual search.

## Introduction

It has long been known that the visual system is limited in its ability to process multiple simultaneous inputs. Psychophysical evidence for visual capacity limitations comes from the observation that irrelevant distractors impair the ability to detect or discriminate a task-relevant target. Distractor interference is of several types. Distractors positioned close to the target can impair target visibility (i.e., the ability to detect the target and distinguish its features, generating the phenomena of lateral masking and crowding) [1,2]. The critical separation for crowding—approximately half the retinal eccentricity—well exceeds visual acuity limits, suggesting that this form of interference arises in higher-order visual processing stages [1]. The “set-size effect” is another form of interference that operates over larger distances in search tasks in which the target does not pop out from the display [3–6]. The set-size effect does not affect target discriminability but increases the time needed to find a target in the presence of a large number of distractors.

Although the set-size effect has been widely documented, its neural substrates have remained unknown. In extrastriate cortical areas, blood-oxygen-level-dependent functional MRI activation decreases as more stimuli are added to a display, suggesting that neuronal populations representing individual inputs engage in mutually suppressive (competitive) interactions [7]. Single-neuron recordings have confirmed the presence of competitive interactions in cortical visual areas V2 and V4, and have shown that attention biases the competition in favor of the attended stimulus while suppressing the effect of distractors [8]. However, these studies have not related neuronal competitive interactions to specific forms of visuo-visual interactions.

Because the set-size effect does not impair discriminability per se, it is thought to reflect a form of attentional, rather than visual, interference. Here we tested this idea by examining how set-size affects search-related neural activity in the lateral intraparietal area (LIP), an area important for spatial attention and eye movements [9]. Monkeys performed a covert visual search task in which they discriminated the orientation of a target surrounded by variable numbers of distractors without shifting gaze to the target. As expected, LIP neurons responded more if the target appeared than if a distractor appeared in their receptive field (RF), thus reliably encoding target location. However, firing rates associated with both the target and the distractors decreased with an increasing number of distractors (set size), reflecting the operation of competitive visual interactions. The set-size-related decline in target responses correlated with performance accuracy and reaction time. The findings suggest that the set-size effect is explained, at least in part, by long-range competitive interactions that limit the strength of signals related to spatial attention.

## Results

### Behavioral Task and Performance

**Figure 1:**
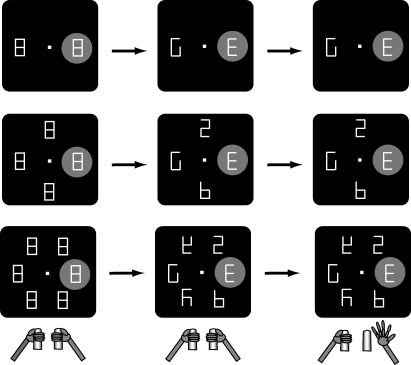
The Covert Search Task The task was run in randomly interleaved trial blocks with set sizes of 2, 4, or 6 elements (top, middle, and bottom rows). A circular array with the appropriate number of figure-8 placeholders remained on the screen in the intertrial interval. To initiate each trial monkeys fixated a central fixation point and grasped two response bars positioned at waist level, outside their field of view (left panels). During central fixation, one placeholder fell in the center of the neuron's RF (gray patch). After a 500 ms presearch period, two line segments were removed from each placeholder, revealing several distractors and one target, an E-like shape (middle panels). Monkeys were rewarded for maintaining fixation and reporting the orientation of the target by releasing the right bar if the “E” was right-facing (right panels) or the left bar if it was left-facing.

Two monkeys performed a covert visual search task in which they discriminated the orientation of a visual target surrounded by a variable number of distractors (Figure 1). A trial began when monkeys shifted gaze to a fixation point located at the center of a stable circular array of 2, 4, or 6 figure-8 placeholders (left panels). The array was positioned so that, when monkeys achieved central fixation, one placeholder (the “RF stimulus”) entered upon a constant location in the center of the RF of the recorded neuron. After a 500 ms delay two randomly selected line segments were removed from each placeholder, revealing a search display with 2, 4, or 6 unique shapes. One of the shapes, a right- or left-facing letter “E” appearing at an unpredictable location, was the search target while the others were distractors. Without breaking central fixation monkeys reported the orientation of the target by releasing grasp of a bar held in the right or in the left hand. We refer to the fixation epoch prior to presentation of the search display as the presearch epoch and to the interval starting with removal of line segments and ending with the bar release as the reaction time or search epoch.

**Figure 2:**
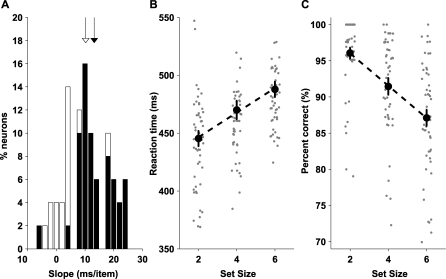
Behavioral Performance (A) Dependence of reaction time on set size in the 50 recording sessions that tested all set sizes. Histogram shows the distribution of slopes representing the dependence of reaction time on set size. Filled bars indicate slopes significantly different from 0. Arrows show the average slope for the entire population (open) and for the significant subset (filled). (B) Dependence of reaction time on set size across the population. Each gray dot is the average reaction time from one session, and the large filled symbols show average and standard error across the sample. The dashed line is the best fit through the data points using Equation 1 (Materials and Methods section). (C) Dependence of accuracy on set size. Each small gray point is the fraction correct in one session (same sessions as in (A) and (B)), and the line shows the best fit to Equation 1 with accuracy as the dependent variable.

To examine the effect of set size, we used interleaved trial blocks in which the stable array contained 2, 4, or 6 elements (Figure 1, top, middle, and bottom rows). Increasing set size was associated with higher reaction times and lower accuracy (Figure 2). The set-size effect on reaction times, estimated using linear regression (Materials and Methods section, Equation 1), was significant in 70% of sessions with an average slope of 10.2 ± 1.1 ms/item (Figure 2A; 13.2 ms/item for the significant subset; both *p* < 0.01 relative to 0). Fitting the population data (Figure 2B) yielded a very similar slope of 10.6 ms/item (confidence interval (CI) [5.8, 15.5]; intercept, 425 ms; regression, *p* < 10^−5^; *R* ^2^ = 0.11). Compared with correct trials, error trials had higher reaction times but a comparable set-size effect (intercept, 459 ms; *p* < 0.05 relative to correct trials; slope 14.7 ms/item; CI [10.2, 17.8]; regression, *p* < 0.05; *R* ^2^ = 0.09). Fitting the accuracy values (Figure 2C) yielded a slope of −2.2%/item (CI [–2.9, −1.5]; intercept, 100.5; regression, *p* < 10^−5^; *R* ^2^ = 0.33). Thus, each additional distractor in the display caused an increase in reaction time of ∼10 ms and a decrease in accuracy of ∼2.2%.

A distinguishing feature of our task is that it required covert attention and a nontargeting motor report (a grasp release) but precluded oriented movement of either eye or limb toward the search target. However, it was possible that even while they maintained central fixation monkeys attempted to shift gaze toward the target. To examine this possibility we measured average eye position in consecutive 100 ms time bins during the search period (0–400 ms after search onset) as well as the end points of the first saccade made within 300 ms after the bar release (when the search array remained on the screen but the fixation point was removed). All eye position measures were uniformly distributed relative to target location (Rayleigh test for directedness of circular distributions, *n* = 1,710, 3,312, and 4,698 trials for set-sizes 2, 4, and 6; *p* > 0.6 for all measures). Thus we found no direct evidence that monkeys tended to shift gaze toward the search target during or after a trial.

### LIP Firing Rates Encode Target Location and Decline with Increasing Numbers of Distractors

**Figure 3:**
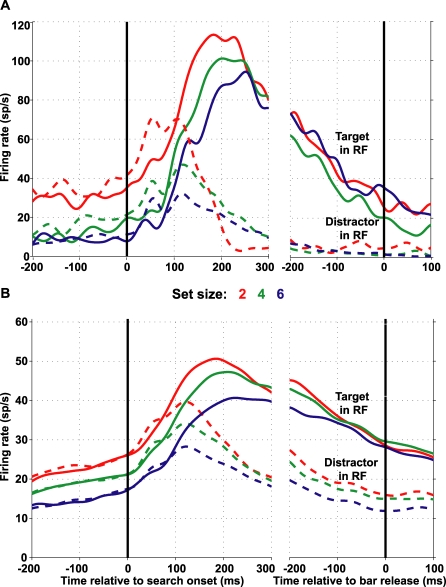
Set-Size Effect in LIP Neurons (A) Response of a representative neuron (neuron 12289), on trials in which the target (solid) or a distractor (dotted) were in the RF, at set-size 2 (red), 4 (green), and 6 (blue). Responses are aligned on search display onset (removal of line segments, time 0) in the left panel and on bar release in the right panel. For display purposes only, spike density histograms were derived by convolving individual spike times with a Gaussian kernel with a standard deviation of 15 ms. Standard error (computed every 1 ms and averaged across all conditions and time bins) was 2.69 spikes/s. (B) Average responses in the sample of neurons tested with all three set sizes (*n* = 50) using the same conventions as in (A). Average standard error was 2.73 spikes/s.

LIP neurons are known to encode target location during visual search, whether search is accompanied by saccades [10–12] or is performed covertly, as in the present study [13]. Accordingly, the neurons that we describe here had robust target location selectivity during the active phase of search (Figure 3). In addition, their firing rates declined as a function of set size.

Figure 3A shows the responses of a representative neuron, and Figure 3B the average responses of the 50 neurons tested at all set sizes. Responses are segregated according to set size (red, green, and blue traces) and according to whether the target or a distractor was in the RF (solid versus dashed traces). During the presearch epoch (left panel, −200 to 0 ms) the visual array was uniform, and the RF visual stimulation was constant across set sizes. Nevertheless, firing rates declined as set size increased from 2 to 4 to 6. Once the placeholders changed shape (time 0 in left panel), neurons showed a small transient response to the visual offset at ∼50 ms latency (see also Text S1, note 1), followed by a robust signal of target location, whereby responses became much stronger if the target was in the RF than if a distractor was in the RF. Both target and distractor responses were lower at higher set sizes, but this neural set-size effect diminished by the time of the bar release (right panels).

### Magnitude and Time Course of the Set-Size Effect

**Figure 4:**
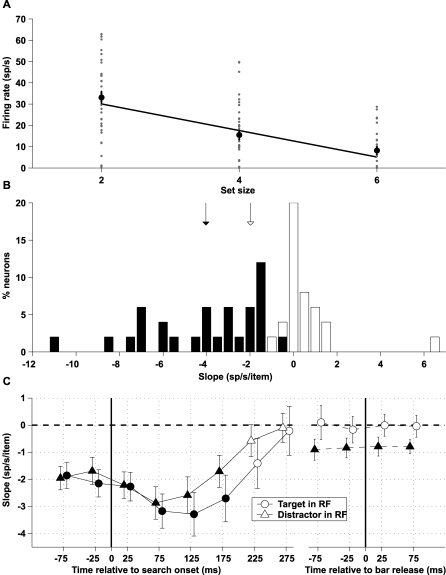
Magnitude and Time Course of the Set-Size Effect (A) Calculation of the set-size effect in the presearch epoch for the neuron shown in Figure 3A. Each point represents firing rate in a correct trial in the 100 ms before search onset. Filled symbols show the mean and standard error, and the line is the best fit linear regression (Materials and Methods section, Equation 2). (B) Distribution of slopes in the presearch epoch (100 ms before bar release) for all 50 neurons. Filled bars show neurons with significant slopes. Arrows show the average for the entire sample (open) and for significant values (56%; filled). (C) Time course of the set-size effect. Each point shows the slope (mean and standard error, *n* = 50 neurons) in 50 ms consecutive bins aligned on search onset (left) or bar release (right). Circles show trials in which the target was in the RF; triangles show distractor trials. Filled symbols show values significantly smaller than 0.

To estimate the magnitude and time course of the set-size effect we fitted firing rates as a linear function of set size using linear regression (Materials and Methods section, Equation 2). We conducted this analysis separately for the presearch epoch (100 ms prior to search onset; Figure 4A and 4B) and in consecutive time bins spanning the search epoch (Figure 4C).

Figure 4A shows the results of the presearch analysis for the example neuron in Figure 3A. Because target location was unpredictable, all trials regardless of target location were pooled for this analysis. The neuron showed a significant set-size effect with a slope of −6.2 spikes/s/item (CI [–7.9, −4.6]; regression, *p* < 10^−11^; *R* ^2^ = 0.31). Across the sample (Figure 4B), 56% of neurons had slopes significantly smaller than 0 with an overall mean of −2.0 ± 0.46 spikes/s/item (−4.0 spikes/s/item for the significant subset; *p* < 0.0001 relative to 0; *n* = 50). Thus, neurons showed a decrease in firing rates of ∼2 spikes/s, on average, for each item added to the display. This effect was present from the beginning of fixation (i.e., from the time when the stable placeholder entered into the RF by virtue of the monkeys' eye movements) (Figure S1).

To follow the evolution of the set-size effect during the search epoch we repeated the regression analysis in consecutive 50 ms time bins, this time segregating trials according to whether a target or a distractor appeared in the RF. Figure 4C shows the average slope as a function of time, in data aligned on search onset (left) and bar release (right), for target and distractor trials (circles versus triangles). In the first 200 ms of search the set-size effect remained comparable to that in the presearch epoch (*p* > 0.2 relative to presearch bins, for each time bin and trial type between 0 and 200 ms after search onset). However, the set-size effect declined markedly thereafter (i.e., slopes increased toward 0), and the average slope became statistically indistinguishable from 0 (open symbols) by 250 ms after search onset for both target and distractor trials (each *p* > 0.05 relative to 0). When the data were aligned on bar release (right) a small residual set-size effect was seen for distractor but not for target trials (all distractor slopes, *p* < 0.03; target slopes, *p* > 0.73 relative to 0). However, no significant differences were found between target and distractor slopes in any time bin (paired *t*-tests, *p* > 0.1). A two-way analysis of variance (ANOVA) with bin and trial type (target or distractor in RF) as factors confirmed that there was a highly significant effect of time (*p* < 10^−10^) but no effect of trial type or interaction between time bin and trial type (*p* > 0.1). The fraction of neurons showing significant slopes reached a peak of ∼55% (60% for the target, 50% for the distractors) between 100 and 150 ms after search onset and dropped to 15% (12% for target, 18% for distractor) in the last 50 ms before bar release. Thus, set-size effects were comparable whether a target or a distractor was in the RF and diminished gradually from the presearch epoch to the time of the bar release. A similar pattern was found in the larger subsets of neurons tested at only two of the three set sizes (Figure S2).

### Magnitude and Time Course of Selectivity for Target Location

Because LIP neurons strongly distinguish between a target and a distractor in the RF, it is important to determine how set size affected neuronal selectivity for target location. We measured target location selectivity using receiver operating characteristic (ROC) analysis, which estimates the probability that an ideal observer can determine whether a target or a distractor is in the RF based on the distribution of firing rates associated with each (see Materials and Methods section). A ROC index of 0.5 indicates no selectivity, while indices above 0.5 indicate preference for the target over distractors in the RF.

**Figure 5:**
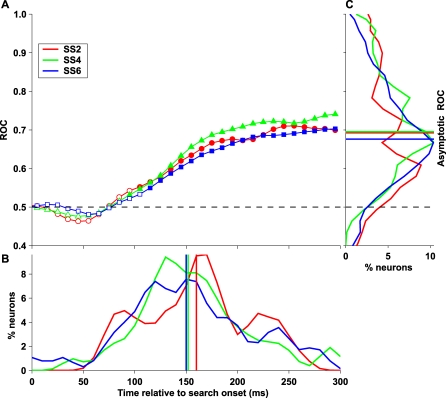
Target Location Selectivity (A) ROC indices reflecting neuronal discrimination between target and distractor for set-size 2 (red circles), 4 (green triangles), and 6 (blue squares). Points show the average indices across the 50 neurons tested at all three set sizes. Filled symbols indicate statistically significant ROC values (*p* < 0.05 relative to 0.5, *t*-test). (B) Distributions of times at which discrimination between target and distractor became reliable on a neuron-by-neuron basis for set-size 2 (red), 4 (green), and 6 (blue). The vertical lines indicate medians. (C) The distribution of asymptotic ROC values calculated as the average ROC values in a 200–300 ms interval after the search onset for each neuron and each set size. Lines indicate medians.

The finding that firing rate versus set size slopes were similar for target- and distractor-related responses suggests that increasing set size reduced firing rates uniformly and thus did not change the difference between target and distractor responses. Indeed, as shown in Figure 5, both the time course and the asymptotic (peak) levels of the ROC values were unaffected by set size. The population ROC value (center panel) became significantly greater than 0.5 at a similar time across set sizes (*p* < 0.05; *n* = 50; 110–120, 90–100, and 130–140 ms for set sizes 2, 4, and 6). Likewise, the distributions of target discrimination times in individual neurons (see Materials and Methods section) showed no effect of set size nor significant differences between set sizes (one-way ANOVA followed by multiple comparisons; median times of 160, 150, and 150 ms for set-sizes 2, 4, and 6). The asymptotic ROC values (measured between 200 and 300 ms after search onset) were also not affected by set size (one-way ANOVA, *p* > 0.1). Thus, increasing set size reduced task-related firing rates but did not reflect the magnitude or time course of target–distractor selectivity.

### Relation to Performance Accuracy

**Figure 6:**
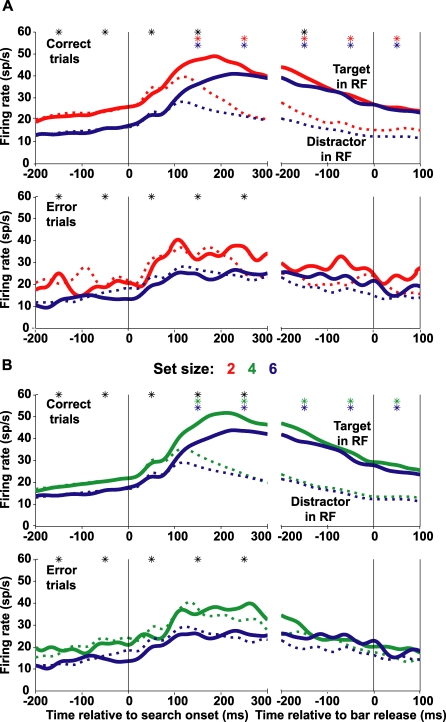
Firing Rates in Correct and Error Trials (A) Average firing rates in correct and error trials, when the target (solid) or a distractor (dashed) was in the RF at set sizes 2 and 6. Data are from 55 neurons that had at least three error trials in each category. The black asterisks (top row) show 100 ms time bins in which firing rates differed significantly between the two set sizes. The red and blue asterisks (next two rows) show 100 ms time bins in which firing rates differed significantly between target and distractors in the RF at the corresponding set size. (B) Same as in (A), for 53 neurons tested at set-size 4 and 6.

To examine the relationship between the LIP response and performance accuracy we analyzed responses on error trials in which monkeys released the wrong bar (Figure 6). Because of the relatively low error rates few neurons had a sufficient number of trials in all trial categories at each set size. Therefore we turned to a pairwise analysis in which we separately compared correct and error trials in subsets of neurons that contributed a sufficient number of error trials at set-size 6 and set-size 2 (Figure 6A, *n* = 55 neurons) and at set-size 6 and set-size 4 (Figure 6B, *n* = 53 neurons). Although a significant effect of set size was present in both correct and error trials (black asterisks indicate *p* < 0.05 in 100 ms time bins), selectivity for target location (difference between solid and dashed traces) was entirely absent on error trials (colored asterisks indicate *p* < 0.05 in 100 ms time bins for the corresponding set size). As discussed in relation to Figure 2, manual latencies in error trials were longer than those in correct trials (for the present subsets of data, 2 versus 6, intercept, 440 ms, slope, 13.8 ms/item; 4 versus 6, intercept, 460 ms, slope, 18 ms/item; all *p* < 0.05 relative to correct trials). However, neuronal target location selectivity was absent up to the time of the bar release (right panels), ruling out the possibility that discrimination may have occurred later on error trials, commensurate with the longer reaction times. These findings suggest that at least some errors reflected failures in target selection, which were associated with a lack of target location selectivity in the LIP.

### Correlation with Reaction Time

Two prior studies have reported that saccade reaction times correlated with the onset of significant neuronal discrimination between target and distractor in the RF [12,14]. However, as shown in Figure 5, in our data neither the time of neuronal discrimination between target and distractors nor the asymptotic level of discrimination varied across set size despite clear effects on reaction time. Indeed, we found no significant correlation between the change in ROC onset times and the corresponding change in reaction time across set sizes (2 versus 4, 2 versus 6, and 4 versus 6; all *r* < 0.02; *p* > 0.3).

**Figure 7:**
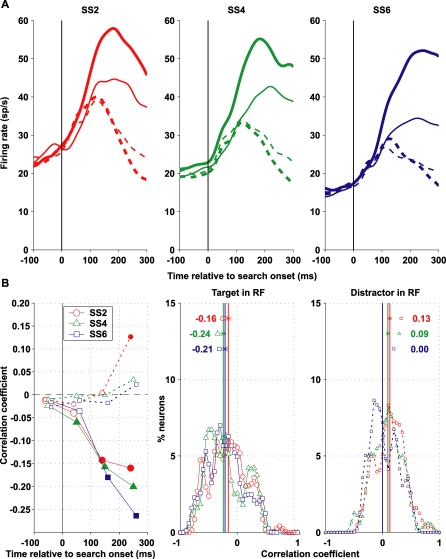
Correlations between Firing Rate and Reaction Time within Each Set Size (A) Population firing rates averaged for trials with responses shorter and longer than the median (thick and thin traces) for the target or distractors in the RF (solid and dashed traces). (B) Correlation coefficients between firing rates and reaction time. Left panel shows the population correlation coefficient computed in 100 ms time bins when the target (solid lines) or a distractor (dashed lines) was in the RF, at set-size 2, 4, or 6. Filled symbols indicate statistically significant values (*p* < 0.05). Middle and right panels show the distribution of coefficients from individual neurons between 200 and 300 ms after search display onset when the target or a distractor was in the RF. Vertical lines and numbers indicate medians; the star represents *p* < 0.05 relative to 0.

In contrast with the constancy in the ROC signal, however, we found that the firing rates associated with the target itself did reliably correlate with reaction time (see also [15,16]). To examine this correlation within a set size, we separated trials into subgroups in which reaction time was shorter (thick traces) or longer (thin traces) than the median for each cell (Figure 7A). Target-related responses had a stronger and faster rise in trials with short reaction times than those with long reaction times, while distractor-related activity showed a much smaller dependence on reaction time. We confirmed these observations by computing trial-by-trial correlations between firing rates and reaction time as a function of time during the trial. To compute the correlation across the population (Figure 7B) we first normalized firing rates and reaction times by subtracting the average in each neuron's dataset. When the target was in the RF population correlation coefficients became significantly negative (indicating that higher firing rates were associated with shorter reaction times) starting 100–200 ms after search onset. In contrast, distractor-related responses were largely uncorrelated with reaction time except for a trend toward a positive correlation, which reached significance only for set-size 2 late in the trial (200–300 ms). The middle and right panels show the distribution of coefficients for target and distractor responses in individual neurons (computed without normalization) between 200 and 300 ms after search onset. Coefficients for the target response were shifted toward negative values with medians of −0.16, −0.24, and −0.21 for set sizes 2, 4, and 6 (all *p* < 10^−5^ relative to 0). Coefficients for distractor responses tended to be positive but had smaller absolute values (0.13, 0.09, 0; *p* < 0.05 for set-sizes 2 and 4). While these analyses included only trials in which the target was in or opposite the RF, we obtained similar results when we included all distractor trials. Thus, variability in the neural response to the target, but much less in that to the distractor, was correlated with variability in reaction time.

**Figure 8:**
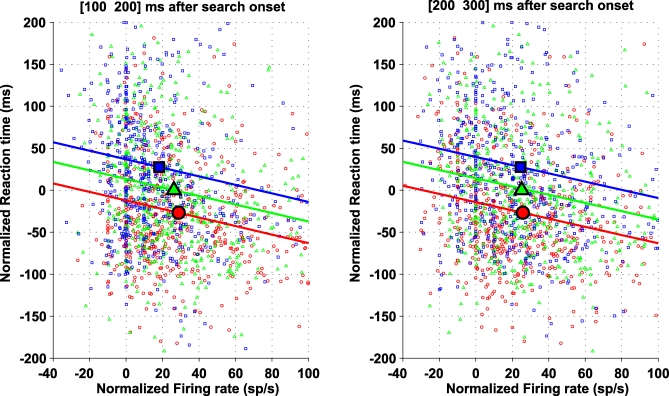
Correlation between Reaction Time and Firing Rates across Set Sizes Each point shows the data from an individual trial (target in the RF), with data from all 50 neurons pooled together. For clarity of presentation only 50% of points (randomly selected) are depicted. Reaction times are normalized by subtracting each neuron's mean reaction time. Firing rates are normalized by subtracting each neuron's average firing rate (−200 to 300 ms after search onset, all correct target and distractor trials at all set sizes), which explains why firing rates are shifted toward positive values. Filled symbols show the average reaction time and firing rates for each set size. Lines are best-fit solutions using ANCOVA (see text).

To see to what extent variability in the target response could account for the set-size effect in reaction time, we fitted reaction times as linear functions of target-related firing rates including set size as covariant (Figure 8; see Materials and Methods section). The analysis yielded slopes of −0.51 ms/spikes/s 100–200 ms after search onset, and −0.49 ms/spikes/s 200–300 ms after search onset, showing that reaction times increased by about 1 ms for each 2 spikes/s drop in neural response. Although modest, these slopes were highly significant (each *p* < 10^−20^ relative to 0). Correlation coefficients were comparable to those in Figure 7 (100–200 ms, −0.21, −0.17, and −0.15 for set-sizes 2, 4, and 6; 200–300 ms, −0.21, −0.17, and −0.15; all *p* < 0.05). The analysis of covariance also showed that intercepts differed significantly across set sizes. Intercepts (measured at 0 spikes/s) at set sizes 2, 4, and 6 were −12, 13, and 37 ms for 100–200 ms, and −14, 14, and 35 ms for 200–300 ms (each *p* < 0.05 for effect of set size). Thus, the analysis revealed two components of the behavioral set-size effect: one, captured by the intercept, was independent of LIP responses while a second, captured by the slope, was significantly correlated with LIP target-evoked responses.

### Competitive Interactions Can Be Triggered by Stimuli outside the RF

In our task visual stimuli were placed at relatively large distances (medians of 15.0° and 10.7° at set sizes 4 and 6; see Materials and Methods section) that exceeded the distances associated with masking or crowding and may also exceed the span of some of the neurons' RF. We wondered if set-size effects represented interactions only within a RF (i.e., if they arose only in neurons that had more than one stimulus in the RF) or whether they represent interactions beyond the border of the classical RF. To address this question we examined whether set-size effects were related to the RF profile as estimated from the memory-saccade task (see Materials and Methods section).

**Figure 9:**
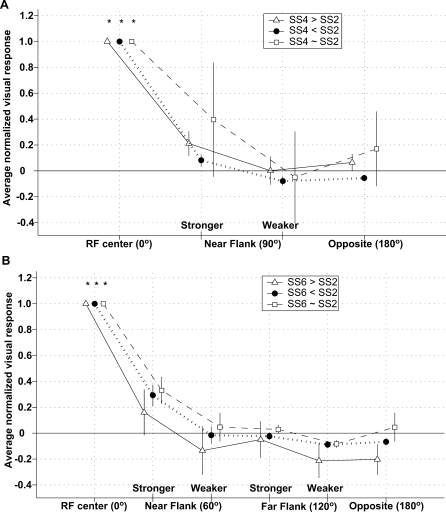
Receptive Field Profiles for Neurons with and without Set-Size Effects (A) Each point shows the normalized visual response (average ± standard deviation) on the memory-guided saccade task at the locations tested with set-size 4 for neurons with different effects of set size. Responses from locations equidistant from the RF center were pooled according to their relative response magnitude (i.e., the location eliciting the stronger or weaker response) regardless of whether they were displaced clockwise or counterclockwise from the RF center. Stars indicate *p* < 0.05 relative to 0. (B) Same as (A) but for set-size 6.

Figure 9 shows the average normalized visual response on the memory-saccade task at the locations used in set size 4 (Figure 9A) and set size 6 (Figure 9B), aligned to the center of each neuron's RF (0°). Because LIP RFs can be asymmetric we sorted the data so that the two locations flanking the RF center were grouped according to their relative response strength (i.e., flankers associated with the stronger and weaker of the two values were averaged separately). Normalized responses were calculated for each neuron by subtracting the baseline firing rate and dividing by the peak response (always in the center of the RF, 0°). Finally, we segregated neurons according to whether firing rates in the 200 ms before search onset were significantly larger, smaller, or equivalent to those at set-size 2 (set-size 4, *n* = 11, 34, and 29, respectively; set-size 6, *n* = 9, 31, and 14).

If competitive interactions were limited to the span of the RF, then neurons with significant set-size effects should have significantly stronger excitatory responses at the stronger flanking location than neurons without a set-size effect. However, this was not the case. Average responses at 90° and 60° flanking locations (set sizes 4 and 6) were not statistically different from baseline (both *p* > 0.1, one-way ANOVA), showing that for most neurons the nearest flanking stimuli fell outside the visual RF [17]. Moreover, response magnitude at flanking locations did not differ between neurons that did or did not have a set-size effect. We also found no consistent tendency for neurons to show inhibitory surrounds near the borders of the RF, as there was no significant dip below baseline at the weaker of the two flanking locations. Activity at the weaker locations was also not related to the set-size effect (*p* > 0.1 for set-size effect, one-way ANOVA). These findings show that competitive effects in the LIP are not straightforwardly predicted by inhibitory surrounds near the excitatory RF and can extend beyond the confines of the classical RF.

### Controls for Manual Response, Reward Expectation, and Target Location Probability

Previously we have shown that during a similar covert search task LIP responses were modified by the active limb, with some neurons having stronger responses to the target if the monkey released the right bar and others preferring left bar release [13]. In the present dataset, approximately one-third of neurons showed limb effects. However, we found that the magnitude and time course of the set-size effect as well as the correlation with reaction time were equivalent in neurons with and without limb sensitivity and, for the former group, were equivalent for responses with the preferred and nonpreferred limbs (Figures S3 and S4). Thus the set-size effect did not depend on limb selectivity.

**Figure 10:**
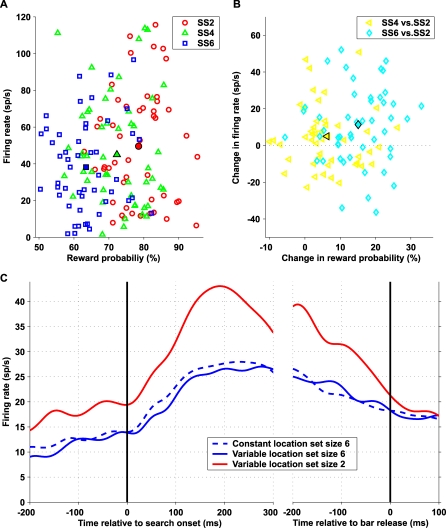
Firing Rates Are Not Correlated with Reward or Target Location Probability (A) Within set-size analysis. Each neuron's average firing rate (100–200 ms after search onset, target in RF, correct trials) is plotted as a function of reward probability in the corresponding trial block. The average firing rates were 49.6 ± 4.4, 44.7 ± 4.3, and 38.2 ± 3.5 spikes/s for set sizes 2, 4, and 6 (*p* < 0.05, one-way ANOVA). Overall correlation was not significant (*r* = 0.11, *p* = 0.2). (B) Across set-size analysis. The difference in average firing rates at set-size 2 versus set-size 4 (triangles), and set-size 2 versus set-size 6 (rhombuses) as a function of the corresponding differences in reward probability. Differences are calculated using data on correct trials in which the target appeared in the RF. The correlation coefficient for the entire dataset was not significant (*r* = 0.0001, *p* = 0.99). The average values ± standard errors for changes in reward probabilities were 6.2 ± 1.3 % and 15.1 ± 1.2 % for set-size 2 vs. 4 and set-size 2 versus 6, respectively; the corresponding changes in firing rates were 5.0 ± 3.2 and 11.3 ± 3.2 spikes/s. (C) Average firing rates (*n* = 10 neurons) when the target was in the RF, for set-size 6 (blue) with 100% (dashed) and 16.7% (solid) location probability, and for set-size 2 (red) with 50% location probability.

A second question is whether the set-size-related decline in activity may have been related to reward probability, which declined together with the monkeys' accuracy [18,19]. To examine this possibility we computed correlation coefficients between session-by-session firing rates and success rate (Figure 10A). We found no correlations either within a set size (Figure 10A; coefficients of −0.09, 0.06, and −0.08 for set sizes 2, 4, and 6; all *p* > 0.58) or in computing the differences across set sizes (Figure 10B; set size 2 versus 4, *r* = −0.12, *p* = 0.06; set size 2 versus 6, *r* = 0.08, *p* = 0.55). We also considered the possibility that monkeys estimated reward probability from local sequences of 10–20 trials rather than globally across long trial blocks [19]. However, correlation coefficients between local measures of firing rate and reward probability (measured in sliding windows of 20 trials) were statistically significant in only 2% of neurons, less than the 5% expected by chance. This precludes the possibility that the set-size effects were due to reward expectation.

Because increasing set size also increases the uncertainty of target location, an important question is whether set-size effects reflected location uncertainty (the diminished probability that the target would appear at any one location) rather than the number of stimuli per se [19,20]. While previous studies reported probability effects in saccade-based tasks [19,20], it is not clear whether monkeys use probability information to adjust the distribution of covert attention. To examine this question we trained monkeys in blocks of trials in which set size was constant (*n* = 6) but target location probability varied between 100% (the target appeared at a single, constant location) and 16.7% (the target appeared with equal probability at each location as in the standard condition). We ran these conditions for 20 sessions for each monkey using long blocks of, on average, 148 trials for each condition (range, 96–358 trials). Despite extensive testing we found no significant differences in either reaction time or response accuracy between the constant and the variable conditions, either in individual sessions or in the pooled data (for reaction time, *p* > 0.1, *n* = 6,003 and 5,994 trials in the constant and variable conditions; for accuracy, *p* > 0.2, *n* = 40 sessions). We also found no differences between reaction time and accuracy in the early and late portions of the blocks (first 25% of trials, constant location, 464 ± 32 ms, 85 ± 6% correct; variable location, 459 ± 71 ms, 83 ± 6% correct; last 25% of trials, constant location, 455 ± 74 ms, 83 ± 5% correct; variable location, 467 ± 56 ms, 83 ± 6% correct). These results suggest that monkeys did not take note of changes in location probability even when these changes were very large (100% to 16.7%). This makes it unlikely that they detected the more subtle changes in probability among set sizes 2, 4, and 6 (50% versus 25% versus 16.7%).

Figure 10C shows responses in a subset of 10 neurons tested at set-size 2 (red traces, 50% probability) and at set-size 6 in 100% and 16.7% probability conditions (blue dashed and blue solid traces). Responses at set-size 2 were much higher than those at set-size 6 but did not differ between probability conditions within set-size 6. A one-way ANOVA in each 100 ms time bin from 200 ms before to 300 ms after search onset revealed highly significant differences between set-size 2 and set-size 6 and either the 100% or the 16.7 % probability condition (*p* < 10^−6^ in each bin) but no significant effect of location probability within set-size 6 (*p* > 0.2 in each time bin). Thus neural responses, like behavioral performance, were related to the number of display elements independent of variations in target location probability.

## Discussion

The visual system does not process visual inputs as isolated entities but fashions integrated representations in which individual objects interact in multiple ways. Although it is known that visual responses in dorsal and ventral extrastriate areas are shaped by competitive visuo-visual interactions, the precise functional consequences of these interactions are not known [7,8,21]. Here we report that competitive interactions also operate in the LIP, a parietal area associated with attention and eye movements, implying that these interactions limit not only the fidelity of visual feature information but also the efficacy of top-down signals of spatial attention. Moreover, competitive interactions in the LIP correlate with the behaviorally measured set-size effect. We discuss our results in the light of prior studies of the LIP and extrastriate cortex and of the neural mechanisms of visual search.

### Covert Attention and Saccade Planning

The LIP has been proposed to encode a priority representation of the visual world, a sparse topographic representation in which only objects that are likely to be attended are strongly represented. Two principal factors are known to activate LIP neurons—the automatic orienting of attention toward a salient but task-irrelevant stimulus [9] and the voluntary selection of a saccade target [11,12,14,15,22–24]. Bisley and Goldberg have shown that presaccadic sustained activity in LIP is related to the deployment of covert attention that precedes an overt saccade [22]. Here we go a step further in linking the LIP with covert attention independently of saccades: in our task neurons were strongly active even though monkeys were explicitly trained to withhold saccades throughout the task. It remains in principle possible that monkeys formed covert plans to make a saccade toward the attended target (although we, like others [25], failed to find direct behavioral evidence for this idea). However, the correlations between LIP activity and performance of the covert search itself strongly implicate this area in covert target selection independent of eye movements. This conclusion is supported by the findings of Wardak et al. that reversible inactivation of the LIP impairs performance on visual search tasks whether these are performed with free or fixed gaze [26,27].

### Contributions to Covert Search

While quantitative models have speculated on the contributions of the LIP to overt saccade decisions [28], accounting for its contributions to covert attention is considerably more challenging. A common working hypothesis is that visual-oculomotor areas such as the LIP and the frontal eye field provide topographic, top-down feedback to feature-selective visual areas including V4, the middle temporal area, and inferior temporal cortex, which boosts visual responses to the attended object [29–31]. The biased competition theory proposes that attentional feedback is especially important in environments containing multiple distractors, where feedback biases neuronal competition in favor of the attended object, allowing neurons representing this object to “win” the competition and filter out the effects of distractors [32].

Our findings are consistent with the idea that LIP plays a specific role in selecting targets and overcoming distractor interference in cluttered visual environments. In our task, as in saccade-based search tasks, neurons selectively encode the location of the search target, and their responses correlate with the efficiency of target selection [11,12,14]. In contrast, responses to nontargets has been shown to correlate with the distracting effects of these objects on performance [22,24,33]. Here we show a novel mechanism of distractor interference: adding distractors to the display suppresses LIP activity through competitive visual interactions, producing a neuronal set-size effect that correlates with the effect of set size on performance. Together with the finding that deficits following LIP inactivation are larger at higher set sizes [26], the findings suggest that the LIP plays a special role in overcoming distractor interference in complex environments.

In light of these considerations, the dissipation of the set-size effect during the reaction time may represent an active process through which the brain suppresses distractor interference. It may be argued that the decline of the set-size effect reflected mere disengagement of the LIP from the task toward the end of the reaction time, as at this time there was a general decline (though not a complete disappearance) in target location selectivity (Figure 3). This possibility is unlikely, however, as high location selectivity was not, in and of itself, necessary for seeing a set-size effect: robust effects were found in the presearch epoch and on error trials, when firing rates were low and there was no location selectivity. Thus, it is more likely that the dissipation of distractor effects reflected an active search-related process. One such process could be selection of the search target. Target-related responses peaked between 100 and 200 ms after search onset, slightly before the time when the set-size effect was filtered out at the population level (200–250 ms). It is therefore possible that the elevated target-related activity suppressed distractor competition, consistent with a biased competition model [8,32]. In addition, feedback about limb motor planning, which reaches the LIP [13], may have helped render responses stereotyped and independent of set size [16].

Because the LIP receives strong input from extrastriate cortical areas, one must consider to what extent the competitive interactions that we report reflect properties of this bottom-up input. However, while competitive effects in extrastriate cortex are based on visual features, those in the LIP are based on spatial location. In areas V2 and V4, the middle temporal area, and the middle superior temporal area, visual competition is triggered when two stimuli are presented in close proximity within an individual RF so that one stimulus has a preferred feature (e.g., orientation or motion direction) while the other is nonpreferred [8,21,34]. These results support a model in which competition (mutual inhibition) arises between neurons with overlapping RFs but different preferred features [8]. In the LIP, in contrast, competitive effects arise among physically identical stimuli (the placeholders during the search epoch) and are triggered even when the competing stimuli are outside the classical RF. Thus competition in the LIP engages neurons that have nonoverlapping RFs but similar (or no) feature selectivity. These considerations suggest that our findings are more closely related with location-based competitive interactions in the superior colliculus and the frontal eye field [35,36] and reflect the internal organization of all three structures in topographic, nonfeature-selective representations. Thus visual clutter appears to affect multiple levels of representation through both space- and feature-based competition.

### Location Uncertainty

Increasing set size in our task also increased the uncertainty about target location or, conversely, lowered the probability that the target appears at any given location. However, our data suggest that under the present conditions monkeys did not explicitly compute and represent location probability. In a control condition in which set size was kept constant but target location probability varied between 100% and 16.7%, we found no effect of location probability on either behavior or neural responses. We note, however, that some models represent the effect of distractors as a broadening of the underlying noise distribution (i.e., distractor-related firing rate distribution) [37]. This type of increase in noise, which may be interpreted as an implicit representation of uncertainty, may indeed be an appropriate mathematical description of our data.

The lack of a probability effect in our task may appear puzzling given prior reports that monkeys are sensitive to manipulations of location or reward probability in saccade-based tasks [18–20]. We suggest, however, that this result is explained by the precise task conditions that we used. Whereas previous studies manipulated the probability of an overt, rewarded saccade, here we used location probability to bias attention, a variable that is by definition covert and cannot be directly rewarded (in our task, reward was linked to a manual response). In addition, the visual conditions in our task provided little incentive to guide attention to the likely target location based on probability information. Because the target was suprathreshold and was present until the manual response, monkeys could simply ignore the prior targets and find the target when it appeared with little loss of accuracy. Indeed, probability effects on covert attention were previously found in monkeys by using brief, near-threshold targets [38] but not in a detection task with suprathreshold stimuli [39]. Thus, our findings do not preclude the possibility that LIP neurons reflect location probability in conditions in which probability is computed and used; they show, however, that neurons are strongly affected by competitive visual interactions independently of target location probability.

### Relation with Performance

The target-related activity in the LIP correlated with both performance accuracy and reaction time. Target location selectivity was entirely absent in error trials, suggesting that many errors reflected failures in locating the target (possibly along with failure in target discrimination or response selection).

In addition, target-related firing rates showed trial-by-trial covariation with reaction time so that higher firing rates were associated with shorter reaction times both within and across set sizes. While the correlation coefficients that we find are modest (−0.2 to −0.3 in Figures 7 and 8), existing evidence suggests that such weak correlations are to be expected in cortical association areas. Because correlations between neural activity and motor output increase along the sensory–motor continuum [40], an area such as the LIP, which represents a nonmotor processing stage, would a priori be expected to covary only weakly with trial-by-trial reaction time. The task that we used is complex and is likely to have engaged multiple areas in addition to the LIP, including extrastriate visual areas and areas related to limb motor planning, procedural memory, motivation, and reward evaluation, all of which have high trial-to-trial variability and weak interneuronal correlations [41–43]. In this regard it is remarkable that the correlations that we report are slightly larger than the average correlation coefficient of −0.09 reported in a saccade-based task [44]. Computational models show that reliable information may be extracted from ensembles of as few as 10–100 task-related neurons with highly variable, weakly correlated firing [42,45], suggesting that our findings reflect significant contributions of the LIP to covert search.

A puzzling aspect of our data is the finding that set size lowered firing rates but did not modify neuronal selectivity for target location (the discrimination between target and distractors in the RF) as indexed by the ROC analysis (Figure 5). We found that increasing set size reduced target- and distractor-related activity by similar amounts, leaving the dynamics of target location selectivity constant across set sizes (Figures 2–5). This appears to be at odds with two prior reports that found a consistent relationship between the time of onset of the ROC and saccade reaction time during visual search [12,14]. It should be noted, however, that variations in ROC dynamics in these studies may have been driven primarily by variations in target-related firing rates, as was the case in the within set-size analysis in Figure 7 (see also [16,44]). Thus, while both the ROC signal and the target response itself can covary with reaction times, the latter may show a more consistent relation across task conditions. Resolving this question will require more detailed understanding how activity is read out from the entire LIP map under different task conditions.

## Materials and Methods

Two adult rhesus monkeys (Macaca mulatta) weighing 8–10 kg (one male and one female) were tested with standard behavioral and neurophysiological techniques. All methods were approved by the Animal Care and Use Committees of Columbia University and New York State Psychiatric Institute as complying with the guidelines within the Public Health Service Guide for the Care and Use of Laboratory Animals. Experiments used standard behavioral and physiological procedures described in detail elsewhere [13,24].

### Single-neuron recording.

Electrode penetrations were aimed at the posterior half of the lateral bank of the intraparietal sulcus as guided by structural MRI. Upon isolation each neuron was first tested with the memory-saccade task on which, after the monkey fixated a central point, a small target annulus (1° diameter) was flashed for 100 ms. After a 1000–1250 ms delay the fixation point was extinguished, and monkeys were rewarded for making a saccade to the remembered location of the target within 100–500 ms. Neural responses were tested at 8–12 locations circularly distributed at a constant eccentricity around fixation, including the location estimated to be the center of the RF (that eliciting the strongest visual response).

The search task was conducted in randomly interleaved blocks of trials. An array containing 2, 4, or 6 figure-8 placeholders remained on the screen from the beginning to the end of a block, including intertrial intervals. A trial (and data collection) began with presentation of a fixation point (a 0.5° red square) at the center of the array. After monkeys grabbed two response bars and maintained central fixation for a 500 ms period, the search display was presented by removing two line segments from each placeholder. The line segments to be removed were selected randomly with the constraints that (1) a single item became the search target (a right- or left-facing letter “E”), (2) each of the remaining shapes was continuously connected, and (3) no shape was presented at more than one location in one trial. The location and orientation of the target were selected randomly with uniform probability and independently of each other. Monkeys were rewarded for continuously maintaining fixation within a window of 2° × 2° and indicating the orientation of the target by releasing the right bar for the right-facing E or the left bar for the left-facing E within 100-1000 ms after removal of the line segments. A correct response was followed by removal of the fixation point and delivery of reward 250 ms later. Incorrect responses (including fixation breaks and early, late, or inaccurate bar releases) were followed by removal of the fixation point without reward delivery. The target array remained on the screen for an additional 500 ms, allowing us to collect eye movement data following bar release. All trials terminated with restoration of the missing line segments, reinstating the placeholder display.

The radius of the placeholder array and its rotation around the center were varied for each training and recording session. During neural recording care was taken that one placeholder fell in the center of the neuron's RF as determined with the memory-guided saccade task. Stimuli were scaled with eccentricity and ranged from 1.5° to 3.0° in height and from 1.0° to 2.0° in width. Median RF eccentricity was 10.6° (range, 2.6–14.3°). Median separations between adjacent array elements at set sizes 2, 4, and 6 were, respectively, 20°, 15.0°, and 10.7° (ranges, 4.0–28.6°, 3.7–20.2°, and 4.6–14.3°, respectively). The ratios between interstimulus distance and eccentricity were 2.0, 1.4, and 1.0 at set sizes 2, 4, and 6, far exceeding the critical ratio of 0.5 that defines the critical distance for crowding [1].

### Data analysis.

Comparisons across samples were made with *t*-tests or paired *t*-tests and one-way and two-way ANOVAs as specified below and evaluated at *p* < 0.05. Regressions were calculated with weighted least-squares algorithms [46]. The 95% CI of the slope and intercept were calculated and used for statistical testing. In addition, we verified the results of the parametric tests using one-way nonparametric ANOVA (the Kruskal–Wallis test) and two-way nonparametric ANOVA (the Friedman test). In all cases the results were equivalent, and, for simplicity, we adopted the convention of reporting only the outcomes of the parametric statistics in the text. In addition, we used ROC analysis, which is a nonparametric measure of the separation between two firing rate distributions and hence the likelihood that an ideal observer can distinguish between the two [47]. Results are shown as mean ± standard error unless otherwise stated.

### Neuronal database.

Data were collected from 107 neurons that had significant spatial selectivity during both memory-saccade and covert search tasks. However, the bulk of the analysis concentrates on 50 of those neurons (24 in monkey 1) that were tested at all three set sizes. Data from additional subsets tested at set-sizes 2 and 4 (*n* = 73), set-sizes 2 and 6 (*n* = 55), and set-sizes 4 and 6 (*n* = 53) were used for the error trial analysis and in additional analyses shown in Text S1.

### Behavior analysis.

Approximately 8% of all trials were discarded from the analysis, as they terminated in fixation breaks or in short- or long-latency bar release. Reaction times were measured as the time from presentation of the search display (measured by means of a light-sensitive diode mounted in the upper left corner of the screen) to the time of the bar release (measured by a transistor–transistor logic pulse emitted upon the onset or termination of contact with the bar). Accuracy was measured as the fraction of correct out of the total number of correct and incorrect bar releases. Reaction times (RTs) were analyzed separately for correct and erroneous responses. To assess sensitivity to set size, reaction times were fit with the regression model

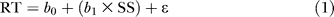
where SS is the set size (2, 4, or 6) and ɛ is random error distributed as multivariate normal (Figure 2A and 2B). The slope *b*_1_ is an estimate of sensitivity to set size in milliseconds per item. This analysis was carried out on a neuron-by-neuron basis, where each data point represented one trial, and across the population, where each data point represents the average RT for a single session. Accuracy (percent correct) was fit using the population data.

### Neuronal analysis.

All analyses were conducted on raw (unsmoothed) spike counts. Firing rates on the memory-saccade task were measured in the baseline (200 ms before target presentation), visual (50–250 ms after target onset), delay (400–900 ms after target onset), and presaccadic epochs (200 ms before saccade onset). A neuron was tested on the search task only if it showed significant spatial tuning during the visual, delay, or presaccadic epochs (*p* < 0.05, one-way ANOVA). Nearly all neurons (96%) had significant spatial tuning during the delay period. Visual responses on the memory-saccade task were highly correlated, across locations, with those during the search task (*r* = 0.94), showing that neurons preserved a constant spatial RF in both tasks.

Several analyses were performed for the covert search task. To measure the sensitivity of firing rates to set size we fit firing rates to the linear model

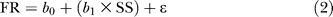
where FR is the trial-by-trial firing rate in the time bin indicated in the text. The coefficient *b*_1_ represents sensitivity to set size in units of spikes per second per item. Fits were obtained separately for trials in which the target or a distractor was in the RF. For the time-course analysis (Figure 4B) our choice of time bin (50 ms nonoverlapping windows) represented the best compromise between the need for temporal resolution and the need to use larger bins for more reliable estimation of firing rates and regressions.

To analyze the relationship between firing rates and reaction time, we first computed the Spearman correlation coefficient between firing rate on a trial-by-trial basis within each neuron (Figure 7B). To compute the coefficient across the population we pooled all trials after normalizing each neuron's data by subtracting the average in the appropriate time bin (Figure 7B, left panel). In a second step (Figure 8), we fit the data using analysis of covariance (ANCOVA), which simultaneously fits separate regression lines of the form

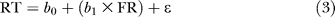
to data from set-sizes 2, 4, and 6. The slope parameter *b*_1_ indicates the sensitivity in units of milliseconds per spike per second, whereas any difference in the intercept indicates the component of RT that depends on set size independently of LIP firing rates. The ANCOVA was computed on normalized data pooled across all neurons. Reaction times were normalized by subtracting the average reaction time across all three set sizes. Firing rates were normalized by subtracting the average neuronal response across all time bins, set sizes, and target/distractor trials.

ROC indices comparing selectivity for target versus distractor in the RF were calculated for each neuron in 10 ms bins aligned on search display onset [47]. We found that ROC analysis was relatively robust to firing rate variations resulting from small bin sizes, allowing analysis with higher temporal resolution. Confidence intervals were obtained by a permutation test with 1,000 repetitions, and a value was deemed significant if its 95% confidence interval did not include 0.5. The onset of significant selectivity was defined as the start of the first four consecutive bins with ROC values significantly different than 0.5 [13].

Each of the above analyses was evaluated for each monkey separately. In no instance did we find significant differences between monkeys, and thus the pooled data are presented throughout the paper. Data are also pooled across right and left bar release; analysis of data segregated according to bar release is included in Text S1.

## Supporting Information

Figure S1 Significant Set-Size Effects Appear Early in the Fixation Epoch Traces show population responses aligned on fixation onset at set-sizes 2, 4, and 6.
(543 KB EPS)

Figure S2 Set-Size Effects in Larger Samples (A and B) The average difference in firing rates between the higher at lower set sizes, in 74 neurons tested at set-sizes 2 and 4 (A) and in 55 neurons tested at set-sizes 2 and 6 (B) when the target (thick traces) or a distractor (thin traces) was in the RF. Each point represents the average and standard error of the differences across the population in 50 ms bins. The asterisks show time bins in which the average difference was statistically nonzero when a distractor (top rows) or when the target (bottom rows) was in the RF.
(C) Fractions of neurons in which firing rates at set-size 2 were significantly higher than at the larger set sizes, evaluated in 100 ms time bins. Line styles indicate the same categories as in (A) and (B).
(857 KB EPS)

Figure S3 Influence of Limb Motor Planning on Set-Size Effects (A and B) Same as in Figure S2A and S2B except that the differences are separated for responses with the preferred hand (left columns) and responses with the not-preferred hand (right columns).
(838 KB EPS)

Figure S4 Correlations between the Target-Related Activity and Search Reaction Time for Preferred and Nonpreferred Limbs (A) Population correlation coefficients in neurons with significant limb effects (*n* = 20 tested at all three set sizes) calculated separately for trials with preferred (black) and nonpreferred (red) manual response. Filled symbols show statistically significant coefficients.
(B and C) The distribution of the correlation coefficients obtained from individual neurons in the interval 200–300 ms after target onset, for both preferred (black) and nonpreferred (red) limbs and for trials with the target (B) or the distractor (C) in the RF. Vertical lines depict median values, and asterisks indicate medians that are significantly different from 0.
(876 KB EPS)

Text S1 Further Details on the Set-Size Effect
In this section we show that (1) the set-size effect was present from the onset of fixation, (2) it was present in a larger subset of neurons tested with only two set sizes, (3) the magnitude and time course of the set-size effect did not differ according to the active limb, even in neurons that showed significant limb modulations, and (4) correlations between activity and reaction times held regardless of the active limb, even for neurons with significant limb preference. We also discuss the small visual onset response to presentation of the search display and the distribution of attention during the presearch epoch.
(42 KB DOC)

## Abbreviations

ANCOVA: analysis of covariance
ANOVA: analysis of variance
CI: confidence interval
FR: firing rate
LIP: lateral intraparietal area
RF: receptive field
ROC: receiver operating characteristic
RT: reaction time
SS2: SS4, and SS6, set-sizes 2, 4, and 6
V1: V2, and V4, cortical visual areas
