# Supplementary material for: Neuronal Correlates of the Set-Size Effect in Monkey Lateral Intraparietal Area
Source: PLoS Biol. 2008 Jul 1;6(7):e158. doi: 10.1371/journal.pbio.0060158 (PMC2443194; doi:10.1371/journal.pbio.0060158)
Supplement: Supplementary file 1 [file pbio.0060158.sd001.doc]

**Text S1**

**1. Alignment on fixation onset**

In our task the placeholder display appeared only once at the beginning of each trial block and remained on the screen throughout that block, including the inter-trial interval. On each trial a stable placeholder entered the RF when the monkey achieved central fixation. Thus, to reveal the response to the stable array we had to align trials to the beginning of fixation. However, we found that on over 70% of trials, the monkeys’ gaze was already at the center of the display when the fixation point appeared – i.e., the placeholder was already in the RF by the time data collection began. To observe the beginning of the visual response we therefore used the remaining 20-30% of trials in which the monkey made a saccade after the fixation point appeared, and the placeholder moved from outside the RF into the RF by virtue of this saccade. **Figure S1** shows the average neural response on these trials (n = 45 neurons with sufficient trials at each set-size). As we expected (Gottlieb, Kusunoki & Goldberg 1998) the response associated with the placeholder’s entrance into the RF was small, since the placeholder was neither salient by virtue of an abrupt onset, nor did it contain a task-relevant target. Nevertheless, a significant set-size effect was seen from the beginning of this response (50-200 ms, p < 0.05, 1-way ANOVA). Thus the set-size effect was visible as soon as the stimulus entered the RF.

**2. The visual transient**

Since in our task the search display was presented through a relatively subtle manipulation – removal of line segments – neurons had a small, barely detectable onset response to this event at about 50 ms after search onset, much smaller than their typical on-response to a flashed object (Oristaglio, Schneider, Balan & Gottlieb 2006). Examination of Figure 3 suggests that this transient was larger when a distractor than when the target appeared in the RF, a difference also seen as a transient dip in the ROC values below 0.5 at around 50 ms (Figure 5). Closer analysis showed that this pattern was seen in only small number of neurons (fewer than 6 at each set-size) and that it was not related to the recent sequence of target locations or target shapes (i.e., location or feature priming). Our best explanation is that some neurons happened to have shape selectivity for some of the distractor patterns (Sereno & Maunsell 1998). However, since we did not store information about the precise distractor shapes used on each trial, we cannot directly examine this possibility. An important point is that the initial visual transient had a comparable sensitivity to set-size as the time bins preceding and following it (Figure 4C).

**3. Set-size effects in larger samples**

In addition to the regression analyses in the main paper, we analyzed set-size effects in larger subsets of neurons that were tested with only two set-sizes. The purpose was to confirm the findings in a larger data set and to verify the results using non-parametric statistics. **Figure S2 (A)** shows the average difference in firing rates between set size 2 and 4 in 74 neurons tested at both set-sizes; and **Figure S2 (B)** shows the differences between set size 2 and 6 in 55 neurons tested at both set-sizes. Differences when the target or a distractor was in the RF are represented by thick and thin traces. Each point represents the average and standard error of the differences across the population. The stars show time bins in which the average difference was statistically above 0 (Kruskal-Wallis test, p < 0.001) when a distractor (top rows) or when the target (bottom rows) was in the RF. (**C**) Fraction of neurons in which firing rates at set size 2 were significantly higher than at the larger set sizes, evaluated in 100 ms time bins. Solid lines represent comparison of set size 2 with set size 4 and dashed lines, comparison of set size 2 and set size 6. Thick lines show target trials and thin lines, distractor trials.

Note that the results are very similar to those shown in main Figure 4B. The firing rate differences of 5 spikes/second between set-size 2 and 4, and 7-10 spikes/second between set-size 2 and 6 in the pre-search and early search epoch are comparable with the slopes of ~2 - 3 spikes/second/item found in the regression analysis; set-size effects are similar for target and distractors; and the effects disappear between 200-300 ms after search onset, but remain slightly higher if a distractor than if the target is in the RF.

**4. Influence of limb motor planning**

Previously we have shown that, while the primary signal in LIP encoded the location of the search target, in a fraction of neurons this response was modulated by limb motor planning (Oristaglio, Schneider, Balan & Gottlieb 2006). The manual release did not independently activate neurons but could modulate the target-related response, so that some neurons responded more or less strongly to the target in the RF if the monkey released the right or left hand. Thus it is important to verify that the set-size effect and the correlations between LIP activity and performance hold regardless of the manual response.

For this analysis we selected neurons that showed a significant limb effect, defined as significant difference firing rates associated with the right and left limb when the target was in the RF at set-size 2, in the 200 ms before bar release (p < 0.05, Wilcoxon rank sum test). To increase the power of our analysis we included all neurons tested with at least 2 set-sizes, and analyzed them as in Figure S2 except that now we separated responses according to the manual release (**Figure S3).** The magnitude and time course of the set-size effect were similar to those in the entire population, and were equivalent for the preferred and non-preferred hands. Included in these plots are neurons with significant limb preference that were tested at set-sizes 2 and 4 (a; 33 neurons) and 2 and 6 (b, n = 25). Similar (but noisier) results were found when the analysis was restricted to those cells tested at all 3 set-sizes (n = 20).

Similarly, the correlations between the target-related activity and search reaction time (calculated as in main Figure 7) held for both preferred and non-preferred limbs (**Figure S4**). Panel **(A)** shows the population correlation coefficients in neurons with significant limb effects (n = 20 tested at all 3 set-sizes) calculated separately for trials with preferred and non-preferred manual response (black and red, respectively). Filled symbols show statistically significant coefficients. Although the data are more noisy due to the smaller number of neurons and trials a clear trend can be seen for an increasingly strong negative correlation and a corresponding shift toward negative values in the distribution of coefficients obtained from individual neurons in the interval 200-300 ms after target onset (b), for both preferred and non-preferred limbs (stars indicate medians that are significantly different from 0). In contrast, the distributions were not significantly different from zero if a distractor was in the RF (c). We also found no difference between the coefficients for the preferred and non-preferred limbs at any set size, whether the target or a distractor was in the RF (all p > 0.07). Thus the correlations between target-related activity and task performance were found regardless of the nature of the manual response.

**5. Spatial attention during the pre-search epoch**

During the pre-search epoch all display elements were identical, and, although monkeys were presumably alert and engaged in the task, it is unlikely that they consistently focused attention on a specific peripheral location. Several considerations support this conclusion. Target location was randomly selected and was not predictable from trial to trial. The fact that we never found higher firing rates when the target than when a distractor was in the RF during the pre-search epoch confirms that monkeys did not anticipate target location (Figure 3B). In addition, the location probability control provides no evidence that monkeys focused attention in advance even when target location was highly predictable. Another possibility is that monkeys adopted an idiosyncratic strategy of focusing on a constant (arbitrary) location before search onset. If this occurred, and if this focusing was necessary for finding a neuronal set-size effect, we should have found set-size effects only in a restricted subset of neurons – neurons with RF overlapping the attended locus. However, in each monkey set-size effects were distributed evenly throughout the visual field, and we found no correlation between the magnitude of the set-size effect and the eccentricity, size or polar direction of the RF (r < 0.01, p > 0.1 in all cases). These findings show that set-size effects could not be accounted for by a specific pattern of spatial attention during the pre-search epoch.

**References**

Oristaglio, J., D. M. Schneider, P. F. Balan and J. Gottlieb (2006). "Integration of visuospatial and effector information during symbolically cued limb movements in monkey lateral intraparietal area." J Neurosci **26**(32): 8310-9.

Sereno, A. B. and J. H. Maunsell (1998). "Shape selectivity in primate lateral intraparietal cortex." Nature **395**(6701): 500-3.
